# The selection of diagnostic modalities in the management of pelvic fracture patients requiring transfers

**DOI:** 10.1186/s13017-015-0027-4

**Published:** 2015-07-30

**Authors:** Chih-Yuan Fu, Shang-Ju Yang, Chien-Hung Liao, Being-Chuan Lin, Shih-Ching Kang, Shang-Yu Wang, Yu-Pao Hsu

**Affiliations:** Department of Trauma and Emergency Surgery, Chang Gung Memorial Hospital, Chang Gung University, 5, Fu-Hsing Street, Kwei Shan Township, Taoyuan, Taiwan

**Keywords:** Pelvic X-ray, Pelvic fracture, Transfer, Sacroiliac joint disruption

## Abstract

**Introduction:**

Pelvic fractures can result in life-threatening hemorrhages. Therefore, pelvic fracture patients must usually be transferred to a trauma center for additional management. We attempted to analyze transferred pelvic fracture patients to determine which diagnostic modalities to use in different treatment settings.

**Materials and methods:**

From May 1, 2008, to February 28, 2014, patients with pelvic fractures who were transferred from other local hospitals within 24 hours after the trauma were enrolled. We compared the pre-transfer conditions and pelvic X-ray results from the local hospitals between the group of patients that underwent further angioembolization at the trauma center and the group that did not. The role of computed tomography (CT) in the decision-making process (i.e., regarding additional angioembolization) at the different institutions was discussed.

**Results:**

In total, 751 patients were enrolled in the current study. Of the patients who received further angioembolization at the trauma center, 77.6 % (121/156) had sacro-iliac (SI) joint disruption on their pre-transfer pelvic X-ray; this rate was significantly higher than that of the patients who did not undergo further embolization (77.6 % vs. 25.5 %, *p* < 0.001). There was no significant difference in the use of pre-transfer CT scans at the local hospitals between the patients who underwent angioembolization and those who did not (53.8 % vs. 50.3 %, *p* = 0.472). Furthermore, of these patients, there was no significant difference in the length of emergency department stay (from arrival to angioembolization) at the trauma center among the patients who underwent pre-transfer CT scans and those who did not (97.4 ± 69.3 minutes vs. 108.6 ± 21.8 minutes, *p* = 0.461).

**Conclusion:**

When managing patients with pelvic fractures, the more attention should be paid to those with SI joint disruption on pelvic X-ray. Because these patients are more likely to require further angioembolization, they should be transferred earlier. Additional CT may be performed after the patient’s transfer to the trauma center to determine subsequent treatment.

## Introduction

Pelvic fractures can result in life-threatening retroperitoneal hemorrhages [1, 2]. The source of the hemorrhage can be divided into three categories: bleeding from the arteries, bleeding from the venous plexus, and bleeding from the cancellous bone itself. These fractures are often associated with a high mortality rate and with chest, abdominal and pelvic-organ injuries [1–3]. The management of patients with pelvic fracture may require angioembolization for hemostasis, an operation room to treat the associated injuries and an intensive care unit (ICU) for close observation [4–6]. Therefore, these patients are usually transferred from a local hospital to a trauma center for further treatment because of the discrepancies between the institutions’ facilities.

During the management of patients with pelvic fractures at local hospitals, the early identification of patients who require further treatment and the early transfer of these patients to the trauma center may be critical. The decision to transfer a patient has historically been based on evaluations with primary tools. Thus, the physicians faced the dilemma of whether to transfer a patient based on limited information. In the evaluation of blunt-trauma patients, primary pelvic X-ray has been usually accepted as an early diagnostic tool and is recommended by the Advanced Trauma Life Support protocol. [7] The pattern of pelvic fracture and the stability of the pelvis can be primarily evaluated with a pelvic X-ray, which remains an essential component of initial fracture and stability screening at many institutions. In the current study, we analyzed these transferred patients to delineate the role of primary pelvic X-rays at local hospitals. In addition, we attempted to identify easily accessible primary pelvic X-ray findings that indicate a need for further treatment according to the primary evaluations in the local hospitals. Furthermore, the selection of the diagnostic modalities used at institutions for patients with pelvic fractures was also discussed.

## Materials and methods

From May 01, 2008, to February 28, 2014, we retrospectively reviewed the trauma registry and the medical records of trauma patients at our institution. Our institution is a government accredited tertiary care center that serves as a major trauma referral center for adjacent counties (over 20 local hospitals). The 64-slice multi-detector computed tomography (CT) scanner is used in our emergency department (ED) as a standard diagnostic tool for trauma patients. Furthermore, in-house attending physicians (trauma surgeons, orthopedic surgeons and interventional radiologists) and appropriate facilities are available to manage patients with pelvic fractures. The operating and angiography rooms are available 24 hours per day, and an angioembolization can be performed within one hour. The hybrid operating room is equipped for unstable patients who require both surgery and angiographic examination.

In our institution, pelvic fracture patients are sent to the angiography room for further intervention based on positive CT scan results (contrast extravasation or large retroperitoneal hematoma). However, there were some rare patients with unstable hemodynamics who did not respond to resuscitation. In the management of such unstable patients, the decision to undergo further hemostasis procedures was based on the results of a primary survey without CT scanning. The patients were sent to the operating room immediately if sonography revealed an intra-abdominal hemorrhage. In contrast, the patients with concomitant pelvic fracture and unstable hemodynamics were sent to the angiography room when other sources of hemorrhage were excluded by primary methods (physical examination, plain film or sonography). In addition, the patients who did not undergo a hemostasis procedure received conservative treatment and close observation in the ward or the ICU. A pelvic circumferential compression device was applied in some cases according to the clinical need and professional judgment [8].

**Figure 1:**
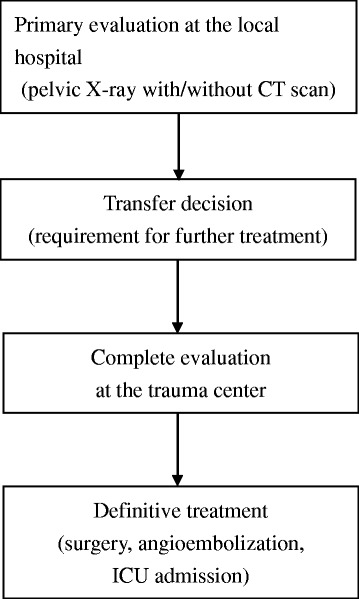
The algorithm for managing transferred patients

The inclusion criteria for the current study were (1) patients aged 18 years or older, (2) patients who were diagnosed with a pelvic fracture (seen on the pre-transfer pelvic X-ray) before transfer, (3) patients who were transferred from other local hospitals within 24 h after the trauma. Pregnant patients, patients with concomitant injuries that required emergency surgery (e.g., thoracotomy, laparotomy) and patients who died in the ED without further treatment or evaluation were excluded from current study. These enrolled patients were generally evaluated and managed as described in Figure 1. Some patients underwent a primary evaluation at the local hospitals with pelvic X-ray, and some underwent abdominal/pelvic CT. After they were transferred to the trauma center, these patients were evaluated again. A definitive treatment decision was then made according to the information obtained from the evaluations at the local hospital, the trauma center or both.

In the present study, we investigated and compared the pre-transfer conditions of the patients who received further angioembolization after being transferred to our trauma center with the condition of those who did not receive further angioembolization. The demographic characteristics, pre-transfer pelvic X-ray results at the local hospitals, Abbreviated Injury Scale (AIS) scores for the pelvis, Injury Severity Scale (ISS) scores, number of blood transfusions, further treatments at the trauma center and outcomes of both groups of patients were routinely recorded. The role of SI joint disruption findings on the pre-transfer pelvic X-ray in the decision to transfer and the need for further angioembolization were analyzed. These two groups of patients were also compared to evaluate the role of CT scans at different institutions in subsequent management and the advantage/disadvantage of pre-transfer CT scanning for the patients who underwent further angioembolization at the trauma center. Furthermore, the difference in the length of ED stay (at the local hospitals or the trauma center) between the patients who underwent pre-transfer CT scans and those who did not was evaluated for the patients who underwent further angioembolization at the trauma center.

In this study, the patients’ images were reviewed retrospectively and blindly by both trauma surgeons and radiologists (all board-certified). The pelvic fracture classification and the patency of the sacroiliac (SI) joint were analyzed. The Young-Burgess classification system was used to evaluate the pelvic fracture patterns. Lateral compression type III, anteroposterior compression types II and III, vertical shearing, and combined-type fractures were defined as unstable, whereas other patterns were considered stable [9, 10]. All of the data are presented as the percentages of patients or as the means with standard deviations. Numerical data were compared using the Wilcoxon two-sample exact test, and nominal data were compared using Fisher’s exact test. All statistical analyses were performed using the SPSS computer software package (version 13.0, Chicago, IL, USA). A value of *p* < 0.05 was considered statistically significant.

## Results

During the 70-month study period, 1,174 patients were admitted to our institution with pelvic fractures. In total, 751 patients (64.0 %, 751/1174) were transferred from other local hospitals for additional evaluation and management. Their demographic information is listed in Table 1. The mean patient age was 42.3 ± 19.8 years. Of these patients, 487 were male (64.8 %) and 264 were female (35.2 %).

**Table 1:** Demographics of the transferred patients with pelvic fractures in current study

Variables	All transferred patients (*N* = 751)
Demographics	
Age	42.3 ± 19.8
Sex (N)	
Male	487 (64.8 %)
Female	264 (35.2 %)
Care level designation of the referring hospitals (number of beds)	
<250	318 (42.3 %)
251-499	389 (51.8 %)
>500	44 (5.9 %)
AIS of torso injuries (scale)	
AIS of the head	3.1 ± 1.1
AIS of the chest	1.4 ± 1.3
AIS of the abdomen	2.5 ± 2.6
AIS of the pelvis	2.7 ± 1.9
ISS (score)	15.6 ± 12.5
ISS <16 (N)	508 (67.7 %)
ISS 16–25 (N)	197 (26.2 %)
ISS >25 (N)	46 (6.1 %)
Y & B classification (N)	
APC	119 (15.8 %)
LC	599 (79.8 %)
VS	19 (2.5 %)
Combination	14 (1.9 %)
Stability of pelvis (N)	
Stable	458 (61.0 %)
Unstable	293 (39.0 %)
SI joint disruption on pre-transfer pelvic X-ray (N)	
Yes	273 (36.4 %)
No	478 (63.6 %)
Post-transfer condition	
SBP on arrival (mmHg)	122.4 ± 81.0
Blood transfusion (ml)	675.4 ± 577.9
ICU admission (N)	
Yes	208 (27.7 %)
No	543 (72.3 %)
Angioembolization (N)	
Yes	156 (20.8 %)
No	595 (79.2 %)
Outcome	
Survival	737 (98.1 %)
Mortality	14 (1.9 %)

Y & B classification = Young & Burgess classification

Values are reported as the mean ± SD

In the present study, 20.8 % (156/751) of the patients received further angioembolization after being transferred to the trauma center, and the other 595 (79.2 %, 595/751) patients received conservative treatment without angioembolization. The patients who received further angioembolization at the trauma center had significantly higher AIS scores for the pelvis (3.9 ± 0.8 vs. 2.4 ± 2.0, *p* = 0.015) and ISS scores (28.8 ± 16.1 vs. 12.2 ± 7.4, *p* < 0.001) than the patients who did not undergo further angioembolization. Furthermore, comparisons of the pre-transfer conditions revealed that the patients who received further angioembolization had significantly more blood transfused (1583.3 ± 877.0 ml vs. 437.4 ± 316.9 ml, *p* < 0.001) and a lower systolic blood pressure (SBP; 94.1 ± 35.6 mmHg vs. 129.8 ± 84.5 mmHg, *p* = 0.004) compared with the patients who did not receive the further angioembolization (Table 2).

**Table 2:** Comparisons of the demographics and pre-transfer conditions between the transferred patients with pelvic fractures who underwent further angioembolization at the trauma center and those who did not

Variables	Transferred patients with pelvic fractures		*p*-value
	Angioembolization (+)	Angioembolization (−)	
	(*N* = 156)	(*N* = 595)	
Demographics			
Age	41.3 ± 20.4	42.6 ± 23.1	1.000^†^
Sex (N)			1.000^‡^
Male	101 (64.7 %)	386 (64.9 %)	
Female	55 (35.3 %)	209 (35.1 %)	
AIS of the pelvis (scale)	3.9 ± 0.8	2.4 ± 2.0	0.015^†^
ISS (score)	28.8 ± 16.1	12.2 ± 7.4	<0.001^†^
Pre-transfer conditions (local hospitals)			
SI joint disruption on pelvic X-ray (N)			<0.001^‡^
Yes	121 (77.6 %)	152 (25.5 %)	
No	35 (22.4 %)	443 (74.5 %)	
SBP (mmHg)	94.1 ± 35.6	129.8 ± 84.5	<0.001^†^
Blood transfusion (ml)	1583.3 ± 877.0	437.4 ± 316.9	<0.001^†^
Role of CT scan			
Pre-transfer CT scans (local hospital)			0.472^‡^
Yes	84 (53.8 %)	299 (50.3 %)	
No	72 (46.2 %)	296 (49.7 %)	
Post-transfer CT scans (trauma center)			<0.001^‡^
Yes	92 (59.0 %)	122 (20.5 %)	
No	64 (41.0 %)	473 (79.5 %)	

Values are reported as the mean ± SD

^†^Wilcoxon rank-sum test

^‡^Fisher’s exact test

The pre-transfer pelvic X-rays showed that 273 (36.4 %, 273/751) patients had SI joint disruption, and 478 (63.6 %, 478/751) patients did not. However, of the patients without SI joint disruption on the pre-transfer pelvic X-ray (N = 478), only seven had an SI joint disruption that was eventually diagnosed with a later CT scan. Furthermore, among the patients who received further angioembolization at the trauma center (N = 156), 77.6 % (121/156) showed SI joint disruption on their pre-transfer pelvic X-ray; this value was significantly higher than for the patients who did not receive angioembolization (77.6 % vs. 25.5 %, *p* < 0.001; Table 2). In other words, the sensitivity and specificity of SI joint disruption findings on pre-transfer pelvic X-rays for evaluating the need for further angioembolization were 77.6 % (121/156) and 74.5 % (443/595), respectively.

Table 2 also shows the rate of CT scan use at the local hospitals or the trauma center for the transferred patients with pelvic fractures who received further angioembolization at the trauma center and for those who did not. There was no significant difference in the rate of pre-transfer CT scan use at the local hospitals between these two patient groups (53.8 % vs. 50.3 %, *p* = 0.472). However, the patients who underwent further angioembolization at the trauma center had significantly higher rates of post-transfer CT scans (59.0 % vs. 20.5 %, *p* < 0.001). Among the patients who underwent further angioembolization at the trauma center (N = 156), those who underwent pre-transfer CT had a significantly longer length of ED stay at the local hospitals (6.8 ± 2.4 h vs. 3.6 ± 3.3 h, *p* = 0.018). In contrast, there was no significant difference in the length of ED stay at the trauma center between the patients who underwent pre-transfer CT and those who did not (97.4 ± 69.3 min vs. 108.6 ± 21.8 min, *p* = 0.461; Table 3).

**Table 3:** Comparisons between the angioembolization patients who underwent a pre-transfer CT scan and those who did not

Variables	*N* = 156		*p*-value
	Pre-transfer CT scan (+)	Pre-transfer CT scan (−)	
	(*N* = 84)	(*N* = 72)	
Age	48.2 ± 31.5	33.3 ± 33.3	0.033^†^
Sex (N)			0.066^‡^
Male	60 (71.4 %)	41 (56.9 %)	
Female	24 (28.6 %)	31 (43.1 %)	
CT at trauma center			<0.001^‡^
Yes	32 (38.1 %)	60 (83.3 %)	
No	52 (61.9 %)	12 (16.7 %)	
Length of ED stay			
Local hospital (from arrival to transfer; hours)	6.8 ± 2.4	3.6 ± 3.3	0.018^†^
Trauma center (from arrival to angioembolization; minutes)	97.4 ± 69.3	108.6 ± 21.8	0.461^†^

Values are reported as the mean ± SD

^†^Wilcoxon rank-sum test

^‡^Fisher’s exact test

Table 4 shows the distribution of the patients who underwent further angioembolization at the trauma center according to the institution at which the CT was performed. Of these 156 patients, seven (4.5 %) did not undergo CT at either the local hospitals or the trauma center. In total, 47 patients (30.1 %) underwent CT only at the local hospitals, and 65 (41.7 %) underwent CT only at the trauma center. CT was performed at both the local hospitals and the trauma center for 37 (23.7 %) patients.

**Table 4:** Distribution of the transferred patients with pelvic fractures who received further angioembolization in the trauma center according to the institution(s) at which the CT scan was performed

Pre-transfer CT scan at the local hospital	Post-transfer CT scan at the trauma center	Patient number (N, %)
-	-	7 (4.5 %)
+	-	47 (30.1 %)
-	+	65 (41.7 %)
+	+	37 (23.7 %)

## Discussion

During a pelvic fracture evaluation, it is usually necessary to transfer the patient to a trauma center for additional evaluation and management if he/she first seen at a local hospital with limited resources. Therefore, the appropriate diagnostic modalities should be carefully selected according to the facilities and resources available at the different levels of institutions.

In the current study, the comparison of the findings on pre-transfer pelvic X-rays taken at the local hospitals revealed that the patients who received further angioembolization at the trauma center had a significantly higher probability of SI joint disruptions on pelvic X-ray compared with the patients who did not receive further angioembolization. (77.6 % vs. 25.5 %, *p* < 0.001; Table 1) For pelvic ring fractures, the literature is quite clear about the importance of posterior injuries (especially SI joint injuries) as a major factor affecting the outcomes [11, 12]. Furthermore, pelvic fractures with SI joint disruption are classified as unstable according to Tile’s systems [13]. Therefore, the SI joint disruption serves as a sign of high-energy impaction that can cause severe injuries and is associated with a high probability that angioembolization will be necessary [14, 15]. The physicians who evaluated these patients at the local hospitals were not familiar with the classification system used for pelvic fractures and were not able to apply it; however, SI joint disruption can be easily observed on a simple plain film of the pelvis. Although it is difficult to predict the need for further angioembolization according to an SI joint disruption on X-ray alone (sensitivity: 77.6 %, specificity: 74.5 %), these patients can be transferred to a trauma center for further evaluation.

In contrast, an increasing number of reports indicate the necessity and importance of CT for evaluating pelvic fracture patients [16, 17]. CT can allow the evaluation of injuries to the intra-abdominal and retroperitoneal organs. Additionally, hemorrhages can be evaluated using the enhanced contrast feature, and the need for further angioembolization can be determined (e.g., in cases of contrast extravasation or large retroperitoneal hematoma) [16–18]. However, there are discrepancies in the staffing and the availability of certain equipment at various institutions. Although CT can provide valuable information, it is not always available at the local hospitals. Furthermore, although hemorrhages were diagnosed with CT at the local hospitals, patients still required transfers to the trauma center for further angioembolization or ICU admission. It has been reported that rapid transport combined with the assessment and management of life threatening injuries reduces morbidity and mortality [19–21]. This “scoop and run” strategy emphasizes fast transfer and a short period of on-site management [22, 23].

In the current study, pre-transfer CT was performed in over half of all transferred patients (51.0 %, 383/751); however, there was no significant difference in the rate of pre-transfer CT scan use between the patients underwent further angioembolization for hemostasis at the trauma center and those who did not (53.8 % vs. 50.3 %, *p* = 0.472). Instead, the patients who underwent angioembolization for hemostasis had significantly higher rates of post-transfer CT use compared with the patients who did not undergo angioembolization (59.0 % vs. 20.5 %, *p* < 0.001; Table 2). In other words, pre-transfer CT scans could not provide sufficient information to determine the need for further angioembolization, whereas the post-transfer CT played a significant role in determining the need for further angioembolization. Furthermore, in the patients who underwent angioembolization for hemostasis, the length of ED stay at the local hospitals, which could not provide subsequent hemostatic procedures, was significantly longer compared with that of the patients who did not undergo pre-transfer CT (6.8 ± 2.4 h vs. 3.6 ± 3.3 h, *p* = 0.018). In contrast, when these patients were transferred to the trauma center, the difference in the length of ED stay between these two patient groups was not significant (97.4 ± 69.3 min vs. 108.6 ± 21.8 min, *p* = 0.461; Table 3). These results indicate that the pre-transfer CT scan could not provide sufficient information to determine the need for further angioembolization and may delay the timing of the transfer. In contrast, with the use of a multi-slice CT scanner in an area with integrated resuscitation and imaging, the CT examination in the trauma center could be performed rapidly while the patient received continuous resuscitation. Therefore, a time-consuming CT scan at a local hospital may not be beneficial for such patients and may not affect the decision to transfer. In contrast, without information from a pre-transfer CT, transferred patients can still be evaluated with rapid CT at the trauma center without delaying the subsequent angioembolization. Thus, the abovementioned results indicate that the primary pelvic X-ray at the local hospitals could be used as a screening tool to determine which patients require transfers. After the primary evaluation and resuscitation, unnecessary examinations are not suggested for the patients who require transfer. Instead, detailed examinations can be performed after the patients are transferred to the trauma center.

In the current study, only seven patients (4.5 %) underwent angioembolization for hemostasis without undergoing a CT examination at the local hospitals or the trauma center. In these cases, persistent hypotension without response to resuscitation was recorded. The patients had no obvious external hemorrhage (of wound or cranio-facial origin) or cavitary hemorrhage (according to chest X-ray or sonography). Therefore, the hypotension may have been caused by a pelvic fracture-related retroperitoneal hemorrhage, which cannot be detected with sonography. Angioembolization could be recommended based on the patient’s hemodynamic instability and pelvic X-ray results. Our previous reports also indicated that pelvic X-ray is valuable for critical patients with an obviously high probability of having a retroperitoneal hemorrhage [24]. Among the patients who underwent angioembolization for hemostasis, 53.8 % (84/156) underwent pre-transfer CT. However, only 56.0 % (47/84) underwent angioembolization on the basis of the pre-transfer CT without undergoing further studies at the trauma center, while 44.0 % (37/84) underwent repeat CTs after being transferred to the trauma center, likely because of the poor quality of the images taken at the local hospital, an inadequate examination (e.g., a non-contrast-enhanced CT scan that could not evaluate the hemorrhage), deteriorated condition after transfer, or incompatibility of the reading system (e.g., the reading software or the device used to store the images). Therefore, CTs at local hospitals are not recommended for patients with pelvic fractures who are scheduled to undergo a transfer. The definitive treatment can be based on post-transfer examinations.

In the current study, patients who were transferred from local hospitals were included in the analysis. These patients may be more critical than the general population. Therefore, the percentage of patients with unstable pelvic fractures or a need for angioembolization was greater than that reported in the published literature [25, 26]. We believe there must have been a few relatively stable patients who were not transferred to our trauma center and therefore were not enrolled in this study. Furthermore, it is difficult to predict patient outcomes or the subsequent clinical pathway on the basis of primary pelvic X-ray results alone because they only provide limited information. Our conclusions may be limited by possible selection bias.

## Conclusions

When managing patients with pelvic fractures, greater attention should be paid to patients with SI joint disruption on pelvic X-ray. Because these patients have a greater likelihood of requiring further angioembolization, earlier transfer is recommended for them. Additional CT to guide decisions about subsequent treatment may be performed after the patient is transferred to the trauma center.
